# Molecularly Imprinted Polymers: Present and Future Prospective

**DOI:** 10.3390/ijms12095908

**Published:** 2011-09-14

**Authors:** Giuseppe Vasapollo, Roberta Del Sole, Lucia Mergola, Maria Rosaria Lazzoi, Anna Scardino, Sonia Scorrano, Giuseppe Mele

**Affiliations:** Department of Engineering of Innovation, University of Salento, via per Arnesano km 1, Lecce 73100, Italy; E-Mails: roberta.delsole@unisalento.it (R.D.S.); lucia.mergola@unisalento.it (L.M.); mariarosaria.lazzoi@unisalento.it (M.R.L.); anna.scardino@unisalento.it (A.S.); sonia.scorrano@unisalento.it (S.S.); giuseppe.mele@unisalento.it (G.M.)

**Keywords:** molecularly imprinted polymers (MIPs), molecular imprinting technology (MIT), molecular recognition, solid phase extraction, sensors, HPLC, drug delivery, catalysis, artificial receptors

## Abstract

Molecular Imprinting Technology (MIT) is a technique to design artificial receptors with a predetermined selectivity and specificity for a given analyte, which can be used as ideal materials in various application fields. Molecularly Imprinted Polymers (MIPs), the polymeric matrices obtained using the imprinting technology, are robust molecular recognition elements able to mimic natural recognition entities, such as antibodies and biological receptors, useful to separate and analyze complicated samples such as biological fluids and environmental samples. The scope of this review is to provide a general overview on MIPs field discussing first general aspects in MIP preparation and then dealing with various application aspects. This review aims to outline the molecularly imprinted process and present a summary of principal application fields of molecularly imprinted polymers, focusing on chemical sensing, separation science, drug delivery and catalysis. Some significant aspects about preparation and application of the molecular imprinting polymers with examples taken from the recent literature will be discussed. Theoretical and experimental parameters for MIPs design in terms of the interaction between template and polymer functionalities will be considered and synthesis methods for the improvement of MIP recognition properties will also be presented.

## 1. Introduction

Molecular Imprinting Technology (MIT) is today a viable synthetic approach to design robust molecular recognition materials able to mimic natural recognition entities, such as antibodies and biological receptors [1–9].

The design of synthetic materials, which are able to mimic the recognition processes found in nature, has become an important and active area of research making in recent years molecular imprinting one of the strategies followed to create materials with recognition ability comparable to the natural systems.

**Figure 1:**
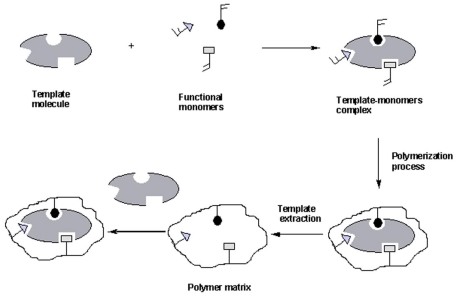
Scheme of molecular imprinting.

MIT is considered a versatile and promising technique which is able to recognize both biological and chemical molecules including amino acids and proteins [10–12], nucleotide derivatives [13], pollutants [14,15], drugs and food [16,17]. Further, application areas include: separation sciences and purification [14,18–23], chemical sensors [24], catalysis [25], drug delivery [26], biological antibodies and receptors system [10,27,28]. MIT is based on the formation of a complex between an analyte (template) and a functional monomer. In the presence of a large excess of a cross-linking agent, a three-dimensional polymer network [29] is formed. After polymerization process, the template is removed from the polymer leaving specific recognition sites complementary in shape, size and chemical functionality to the template molecule (Figure 1). Usually, intermolecular interactions like hydrogen bonds, dipole–dipole and ionic interactions between the template molecule and functional groups present in the polymer matrix drive the molecular recognition phenomena. Thus, the resultant polymer recognizes and binds selectively only the template molecules.

The main advantages of molecularly imprinted polymers (MIPs) are their high selectivity and affinity for the target molecule used in the imprinting procedure. Imprinted polymers compared to biological systems such as proteins and nucleic acids, have higher physical robustness, strength, resistance to elevated temperature and pressure and inertness towards acids, bases, metal ions and organic solvents. In addition, they are also less expensive to be synthesized and the storage life of the polymers can be very high, keeping their recognition capacity also for several years at room temperature.

The scope of this review is to provide a general overview on MIPs field, some significant aspects about preparation and application of the molecular imprinting polymers with examples taken from the recent literature will be outlined. The discussion will evaluate some theoretical and experimental parameters for MIPs design in terms of the interaction between template and polymer functionalities, and synthesis methods for the improvement of MIP recognition properties. In the present review various MIPs applications will also be considered focusing on more recent separation techniques, sensors and biosensors, catalysis and drug delivery.

## 2. Molecular Imprinting Process

As reported above, molecular imprinting is a technique to synthesize highly cross-linked polymers capable of selective molecular recognition. The principle is summarized in Figure 1: polymerization of a monomer occurs in the presence of the target molecule (template) which is incorporate in the polymer matrix. The process starts with dissolution of template, functional monomer, cross-linking agent and initiator in a porogenic solvent. Functional monomers are chosen to interact with the template molecule since the formation of a stable template-monomer complex is fundamental for the success of molecular recognition. Monomers are positioned spatially around the template and the position is fixed by copolymerization with cross-linking monomers. The polymer obtained is a macroporous matrix possessing microcavities with a three-dimensional structure complementary to that of the template. Thus, the removal of the template molecules from the polymer, by washing with solvent, leaves binding sites that are complementary in shape to the template. Consequently, the resultant polymer recognizes and binds selectively the template molecules. The binding sites show different characteristics, depending on the interactions established during the polymerization. Traditionally, molecular imprinting is classified according to the nature of the interactions between monomer and template during the polymerization.

For their insoluble nature, MIPs are notoriously difficult to characterize. However, some analytical techniques can be utilized for their chemical and morphological characterization, including solid-state NMR techniques [30], elemental micro-analysis and Fourier-Transform Infrared Spectroscopy (FT-IR) [31] which can be applied to obtain chemical information on the composition of the polymer. CP-MAS (cross polarization magic angle spinning) NMR spectra for MIP characterization have been reported in a few works even if solid state NMR is a useful method to determine chemical composition of insoluble polymers. For instance, Annamanna and co-workers [30] very recently confirmed the incorporation of crosslinking agent EGDMA and functional monomer 4-vynilpyridine in the polymer backbone of the prepared MIP by ^13^C CP-MAS-NMR. More often FT-IR studies have been used for MIPs characterization through the evaluation of functional groups incorporation in the polymer and through the comparison between polymer and functional monomer spectra to follow the decreasing or disappearance of some signals, such as carbon double bond stretching of a vinyl functional monomer, as a result of polymerization of the polymerizable group of the monomer [31].

Morphological characteristics of MIPs can be properly investigated by microscopy techniques, such as light microscopy to verify the natural integrity of polymer beads and scanning electron microscopy (SEM) to image polymer macro-pores [31,32]. Moreover, nitrogen sorption porosimetry by using BET (Brunauer, Emmett and Teller) analysis is the main technique used to determine specific surface area, specific pore volume, pore size distribution and average pore diameter values of the polymer particles while mercury intrusion porosimetry is sometimes used and is more suitable for larger pores characterization.

However, a very important level of characterization for MIPs concerns the molecular recognition behavior, such as the binding capacity, and one of the best methods for evaluating binding capacity and selectivity is the batch rebinding. MIPs can also be used directly as chromatographic stationary phases which generally provide a quicker and easier method for analyzing the binding properties of MIPs [22,32,33]. Scatchard analysis is a common model to evaluate the binding behavior of MIPs in batch rebinding experiments. Typically non-covalent binding between template and functional monomer gives a Scatchard plot with two straight lines indicating heterogenous affinities of the binding sites in the polymer that are calculated with two association constants corresponding to the high and low-affinity binding sites [34]. On the other hand, Freundlich and Langmuir-Freundlich (LF) isotherms represent most suitable chromatography theoretical models for characterizing and understanding chromatographic features of MIPs columns. These methods, which are also called continuous heterogeneous binding models, can well characterize MIPs by calculating total number of binding sites, heterogeneity index and mean binding affinity. Among them, the LF isotherm is the most commonly model used for characterizing MIPs as HPLC stationary phases prepared by either covalent or non-covalent imprinting [33].

An even better MIP evaluation than imprinting factor seems to be the selectivity for a different molecule, but structurally related to the template, because often the presence of the template during polymerization synthesis causes general changes in the morphology of the polymer, such as porosity and surface area, which are confirmed from binding differences compared with the polymer control that may not be exclusively due to the presence of imprinted sites. On the other hand, the imprinting factor must be considered along with selectivity results since a better or less good retention of a certain compound over another one may just be due to their particular physicochemical properties rather than to specific imprinted sites.

### 2.1. Prepolymerization Studies

Good interactions between monomer and template represent a preliminary essential condition to obtain MIP networks with potential recognition sites. Today there are two main strategies employed for MIP technology depending on the nature of prepolymerization interactions between template and monomer:

- self-assembling approach [35], similar to the biological recognition systems, that uses non-covalent forces, such as hydrogen bonds, Van der Waals forces, ion or hydrophobic interaction and metal-coordinations;
- preorganized approach [2], which uses covalent reversible bonds giving a rather homogeneous population of binding sites and reducing the non-specific sites. However, to remove the template from the polymer matrix, it is necessary to cleave the covalent bounds.

Amongst these, self-assembling is still the most frequently adopted approach for the preparation of MIPs due to the simplicity of complex formation and dissociation and the flexibility in terms of available functional monomers that can interact with almost any kind of templates. On the other side, the related MIPs have lower binding affinity than those prepared using covalent methods. Hwang and Lee [36] prepared, by using a bulk technique, cholesterol-imprinted polymers either in a covalent and a non-covalent approach and they compared MIPs performances as stationary phases for HPLC columns. They found less peaks broadening, higher adsorption capacity and about fivefold higher chromatographic efficiency for the covalently imprinted polymer in comparison with non-covalently imprinted polymers.

Various works reported in the literature demonstrated that prepolymerization studies on self-assembling systems can be useful for the selection of suitable functional monomers and solvents for specific template molecules. The formation of functional monomer-template complexes has been investigated by spectroscopic and theoretical approaches, that includes UV-Vis and NMR spectroscopies and theoretical models as well [37–43].

Job’s method, titration curves and binding isotherms are currently used to determine the nature of the interactions, the coordination number of the complex monomer-template and the association constant by using spectroscopy techniques. For example, the formation of the complex between nitrofurantoin with 2,6-bis(methaacrylamido) pyridine (BMP) was studied by NMR spectroscopy revealing a hydrogen bonding interaction between the imide moiety of nitrofurantoin with the protons of the pyridine moiety of BMP and allowing also the calculation of the association constant. Similar studies were also reported between carboxyphenyl aminohydantoin (CPAH) and 2,6-bis(methacrylamido) pyridine (BMP). Titration curves were performed and the binding constant was calculated using nonlinear curve fitting for a 1:1 complexation model and the complex stoichiometry was independently confirmed by a Job plot method [44].

**Figure 2:**
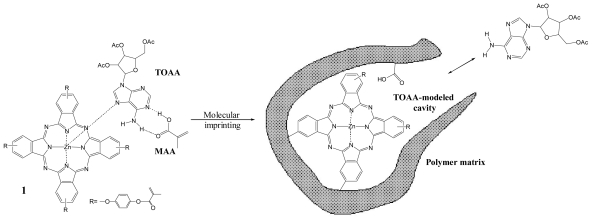
Schematic representation of molecular imprinting of tri-*O*-acetyladenosine (TOAA) using Zn(II) tetra(4′-methacryloyloxyphenoxy)phthalocyanine **1** and methacrylic acid (Adapted from [27]).

Longo *et al.* in 2006 [45] evaluated the binding affinity and selectivity of a new phthalocyanine, as potential monomer towards nucleoside derivatives, by using UV-vis titration experiments. The experiment allowed the calculation of the association constant Ka, determined by the modified Benesi-Hildebrand equation, of a zinc phthalocyanine with tri-*O*-acetyladenosine (TOAA). The calculated value was 1.35 × 10^4^ M which confirms potential use of the zinc phthalocyanine as functional monomer in the formation of molecularly imprinted polymers for nucleoside receptors. Successively, Longo *et al.* [27,34] prepared selective MIPs having the phthalocyanine-based recognition centre as receptors for tri-*O*-acetyladenosine (TOAA) or for RNA nucleoside (Figure 2).

**Figure 3:**
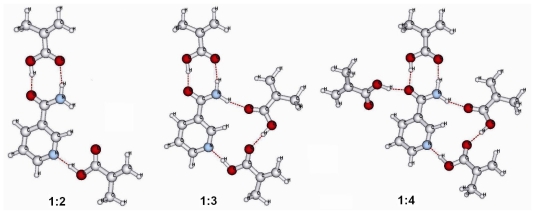
The most stable prepolymerization complex structures for a ratio of 1:2, 1:3 and 1:4 between nicotinamide and methacrylic acid (Adapted from [40]).

In 2009 Del Sole *et al.* [40] reported the studies of prepolymerization interactions between nicotinamide and methacrylic acid in chloroform and acetonitrile by using ^1^H-NMR spectroscopy. The results of this work suggested a possible interaction between nicotinamide and methacrylic acid mainly based on hydrogen-bonding formation between amide protons of template and methacrylic acid. Moreover, computational Density Functional Theory (DFT) studies on the complex (Figure 3) and solvent allowed a better understanding of hydrogen-bonding interactions.

Wei *et al.* [38] explored the potential use of Molecular Dynamics (MD) simulations for selecting the most suitable monomers for 17β-estradiol which was used as model template. Hydrogen-bonding strength was evaluated and the results agreed with previously reported results on batch rebinding experiments. Moreover, experimental ^1^H NMR titration studies confirmed the theoretical results.

In another work [41], a computational screening of 18 monomers, commonly used, that are able to interact with cholic acid (the template) was used to rapidly select the most suitable monomers for synthesizing cholate-imprinted and non-imprinted polymer networks. However, since the modeling is performed using some approximations, differences can occur between modeling and experimental results, especially when polymerization and rebinding steps are done in different liquids.

Pietrzyk [42], using DFT energy optimization calculations, visualized the most stable MIP-melamine complex as triprotonated melamine template with three prepolymerized bis(2,2′-bithienyl)-benzo-[18-crown-6]methane monomers.

Finally, in recent works it was demonstrated that all components (template, functional monomer, solvent, initiator, cross-linker) in a prepolymerization mixture can affect template complexation [39,43]. For instance, molecular dynamics simulations of bupivacaine template in a typical prepolymerization system were performed and the template-methacrylic acid complexation, the role of chloroform and ethylene dimethacrylate in presence of the initiator, were evaluated in conjunction with ^1^H NMR spectroscopy experiments, in order to argue the heterogeneity observed in MIPs [39].

### 2.2. Optimization of MIPs Synthesis

In the synthesis of MIPs, many parameters have to be assessed since they can influence morphology, properties and performance of the polymers. Even if many authors have tried to investigate and understand the role of different parameters in MIPs preparation, a rational comprehension of all of them is still quite difficult to achieve and represents a critical point in MIP field; however, some remarks in MIPs synthesis can be highlighted [8].

Today, the most common method used to obtain MIPs is the free radical polymerization. Generally, the synthesis procedure is performed under mild reaction conditions (e.g., temperature lower than 80 °C and atmospheric pressure) in bulk or in solution, and it is tolerant for a wide range of functional groups and template structures. The polymerization reaction is normally very rapid; it is started by an azo-initiator, commonly azo *N*-*N*′-bis isobutyronitrile (AIBN) and by thermal or photochemical initiation. Data in literature demonstrate that photo-initiated polymerization at low temperature decreases the kinetic energy of the prepolymerization complex increasing its stability and allowing greater binding capacity and specificity than thermal initiated polymerization which requires temperatures higher than 40 °C [16,44,46]. Recently Athikomrattanakul *et al.* [44] attempted to prepare MIPs for nitrofurantoin recognition by thermal initiation, but was unsuccessful. The author, according to the assumption of other papers [16,46], used a photo initiation at low temperature (4 °C) with Irgacure 127 as initiator to synthesize MIPs by using a non-covalent approach. Thus, two different polymers were obtained with carboxyphenyl aminohydantoin as template and DMSO/acetonitrile (67/33) as the porogen and for these polymers interesting imprinting factors for carboxyphenyl aminohydantoin (3.38 and 3.53) and also for nitrofurantoin (2.27 and 2.49) were calculated.

In the synthesis of MIPs, the choice of the chemical reagents is of primary importance in order to obtain efficient functional MIPs. A wide range of template molecules such as drugs, amino acids, carbohydrates, proteins, nucleotide bases, hormones, pesticides and co-enzymes have been successfully used [10–17].

**Figure 4:**
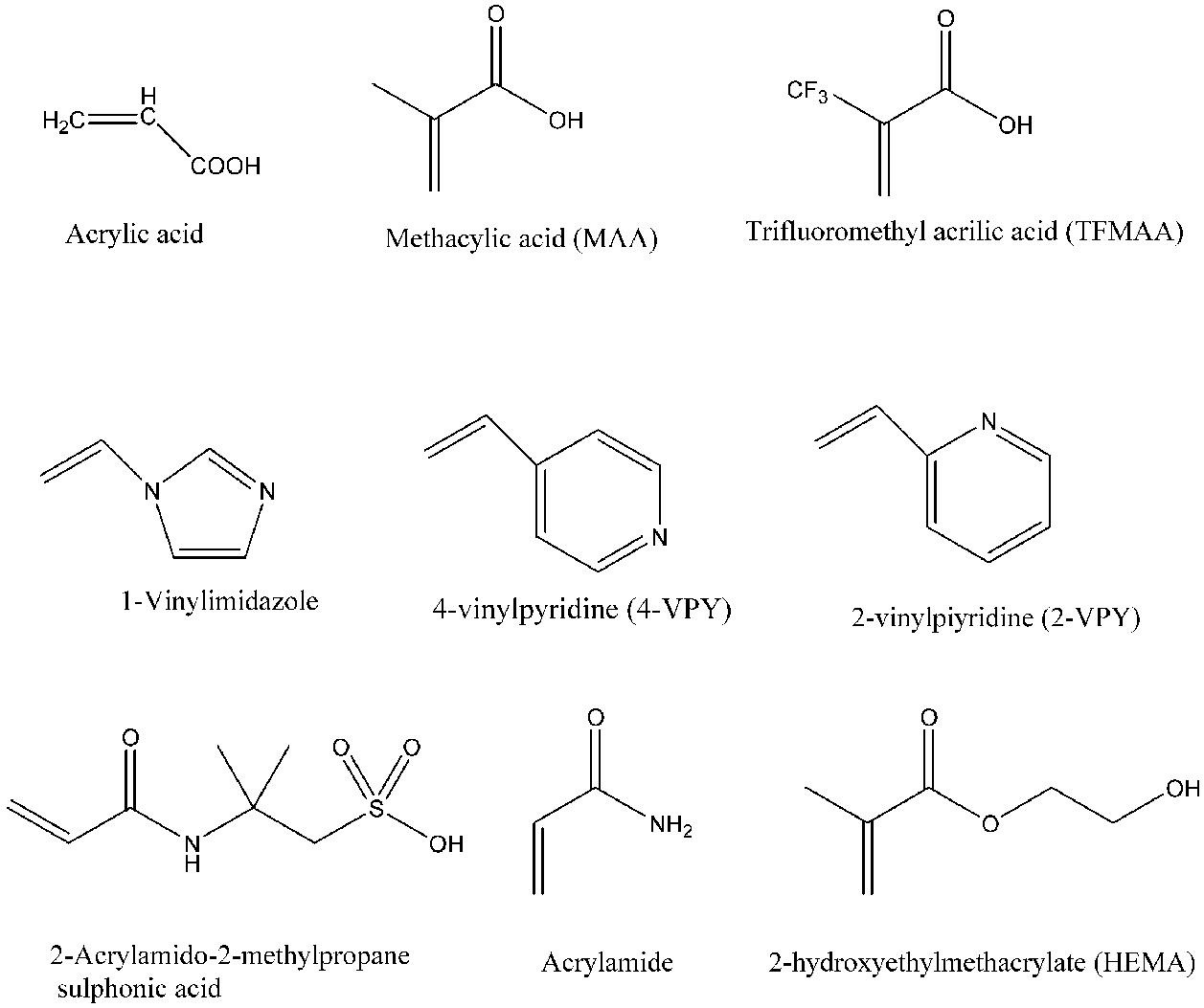
Structure of the most common monomers used for molecular imprinting.

The choice of monomer is very important in order to create highly specific cavities designed for the template molecule. Typical functional monomers (Figure 4) are carboxylic acids (acrylic acid, methacrylic acid, vinylbenzoic acid), sulphonic acids (2-acrylamido-2-methylpropane sulphonic acid), heteroaromatic bases (vinylpyridine, vinylimidazole). In the non-covalent approach they are normally used in excess compared to the template to favor the formation of template-monomer assemblies. In fact, association between the monomer and the template is governed by an equilibrium, and the functional monomers normally have to be added in excess, relative to the number of moles of the template to favor the formation of the complex, with template:functional monomer ratios typically of 1:4. Consequently, this led to a number of different configurations of the template-functional monomer complex, which produced a heterogeneous binding site distribution in the final MIP, with a range of affinity constants. The extensive use of methacrylic acid (MAA) is due to its capability to act both as hydrogen bond and proton donor and as hydrogen bond acceptor. Moreover, more strong functional monomers were developed via metal coordination interactions to bind specific amino acid sequences [47]. New functional monomers, based on polymerizable amidines and ureas, have been developed for stoichiometrically imprinted polymeric receptor of β-lactam antibiotics, reducing non-specific adsorption [48]. The best monomers for synthesizing imprinted materials are selected considering strength and nature of template-monomer interactions as discussed in the previous paragraph.

**Figure 5:**
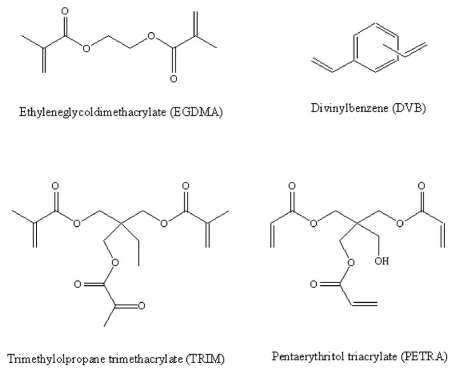
Structure of the most common cross-linkers used for molecular imprinting.

In imprinted polymers synthesis, the cross-linker also fulfils important functions. The cross-linker is important in controlling the morphology of the polymer matrix, serves to stabilize the imprinted binding sites and imparts mechanical stability to the polymer matrix in order to retain its molecular recognition capability [49]. Different cross-linkers have been used (Figure 5). High cross-link ratios are generally used in order to access permanently porous (macroporous) materials with adequate mechanical stability. Ethylene glycol dimethacrylate (EGDMA) and trimethylolpropane trimethacrylate (TRIM) are the most commonly employed. Some authors found that cross-linker has a major impact on the physical characteristics of the polymers and much less effect on the specific interactions between the template and functional monomers [37,50,51]. TRIM as cross-linker gives polymers with more rigidity, structure order and effective binding sites than EGDMA. In the case of polymerization obtained by precipitation method, it has been seen that optimizing the amount of cross-linker and reducing the concentration of the template, the polymer binding properties are improved and the level of non-specific interactions is decreased [52]. In another study it has been observed that the type of cross-linker strongly influences the final size and yield of MIP nanoparticles [53]. In fact, when divinylbenzene was used as the cross-linker, polydisperse MIP particles were obtained in low yield, whereas, trimethylolpropane trimethacrylate (TRIM) led to uniform nanoparticles in high yield (90%).

Moreover, nature and volume of the solvent play also an important role in the molecular imprinting process. The most common solvents used for MIPs synthesis are toluene, chloroform, dichlorometane or acetonitrile. The solvent serves to bring all the components (monomer, template, initiator, cross-linker) into one phase in the polymerization and it is responsible for creating the pores in macroporous polymers. The solvent should produce large pores to assure good flow through properties of the resultant MIP and increasing the volume of the solvents, the pore volume of the polymer enlarges. For this reason it is quite common to refer to the solvent as the “porogen”. The porogen in a non-covalent imprinting polymerization should also be chosen considering its role in promoting template-functional monomer complex formation: less polar solvents such as chloroform or toluene increase the complex formation facilitating polar non-covalent interactions such as hydrogen bonds; more polar solvents tend to dissociate the non-covalent interactions in the prepolymer complex, especially protic solvents that afford a high degree of disruption of the hydrogen bonds. However in some papers efficient MIPs have been prepared in rather polar solvents (e.g., acetonitrile/water or methanol/water) since strong template-monomer interactions have been observed. For instance, a water-compatible imprinted polymer with strong affinity for a polar 1-methyladenosine template has been prepared in acetonitrile/water 4/1 and successfully used as solid phase extraction (SPE) sorbent for the analyte extraction from spiked human urine samples [54].

Usually apolar, non-protic solvents, such as toluene or chloroform, are preferred in MIPs synthesis whereas, if hydrophobic forces are involved in the complexation process, water or other protic solvents could be selected as well. Moreover, it was observed that, after the polymerization, the rebinding performance is optimized when carried out in the same solvent used for the imprinting, suggesting that the optimum rebinding conditions require the same or very similar solvation conditions used for the polymerization.

### 2.3. MIPs Physical Forms and Methods

MIPs can be prepared in a variety of physical forms, using different method, depending on their final application [9,20]. The conventional approach is to synthesize MIPs in bulk, then grind the resulting polymer and successively sieve the particles into the desired size ranges according to the specific application. This method is the most popular since it is simple; nevertheless, crushing, grinding and sieving to obtain the appropriate particle sizes is tedious and time-consuming and often produces particles that are irregular in size and shape. In addition, some interaction sites are destroyed during grinding, reducing the MIP loading capacity and, since only a portion of the original polymer is used, this method suffers from high consumption of the template molecules. Most imprinting publications are still based on use of this method although the scarce control of the MIP physical form and difficulties in scaling up MIP production are important drawbacks. In order to overcome these problems, alternative methods to prepare novel MIP formats, such as MIP beads, membranes, *in situ* prepared monoliths, surface imprinting, molecularly imprinted monolayers have been developed in recent years [9].

Regular beads can instead be obtained using precipitation polymerization method. This technique allows the formation of imprinted beads with the same reaction mixture used in the bulk method except for the presence of a higher amount of porogen. Polymer chains will continue to grow, precipitating only when become large enough to be insoluble in the reaction mixture. Then the polymer beads are easily recovered by washing and centrifugation operations. This technique is easy, less time consuming than bulk polymerization and provides regular beads in good yields. The particles diameters decrease, increasing the porogen volume probably due to the formation of less oligomers and nuclei under diluted conditions and with an increase of flow ability of the polymer, thus less monomers and cross-linkers diffuse to the surface and polymerize into the small particles. In a recent work [53], a feasible defined control of MIP size from nanoparticles to microbeads that can be utilized in different applications by changing the reaction conditions was obtained. The change of particle size, while maintaining excellent recognition property, was achieved by varying the ratio of two different cross-linking monomers (DVB and TRIM) in similar precipitation polymerization conditions. Polymers in the range of 130 nm to 2.4 μm covering all pore sizes typically obtained with precipitation technique were prepared. Mosbach and Mayes [55] prepared spherical beads in liquid perfluorocarbon by suspension imprinting polymerization in the presence of a stabilizer and a surfactant. More recently Zourob *et al*. [56], using a polycarbonate-based spiral microflow reactor, demonstrated that in mineral oil, which is less expensive than perfluorocarbon, it is not necessary to add any stabilizer or surfactant to produce monodisperse MIP beads.

In recent years molecularly imprinted polymer membranes have attracted significant interest. Porous free-standing molecularly imprinted polymer membranes were synthesized by the method of *in situ* polymerization using the principle of synthesis of interpenetrating polymer networks and tested in solid-phase extraction of triazine herbicides from aqueous solutions. Addition of oligourethane acrylate provided formation of the highly cross-linked MIP in the form of a free-standing 60 μm thick flexible membrane [57]. However, for separation purposes low membrane permeability was still the main obstacle for their application in separation processing. Yang *et al*. [58] prepared molecularly imprinted nanotubes supported by a porous alumina membrane. The imprinted nanotubes could be used directly for the separation of biomolecules, without the alumina support being removed.

Monolithic MIPs have also been prepared by a simple, one-step, *in situ* free-radical polymerization process directly within the confines of a chromatographic column without the need of grinding, sieving and column packing.

Surface grafting of MIP layers onto preformed beads has also been proposed as a technique to obtain chromatography-grade imprinted polymers. In this method, thin imprinted layers have been used as coatings on chromatography-grade porous silica or spherical polymers using several techniques to retain the radical polymerization at the surface of the beads.

Molecularly imprinted monolayers have also been prepared for sensor applications. Lahav *et al*. produced recognition sites for a naphthacenequinone derivative in a thiol monolayer on gold electrodes through a photochemical imprinting method [59]. The group of Piletsky and Turner developed a MIP sensor for domoic acid (DA) based on amino-substituted methacrylate crosslinked with ethylene glycol dimethacrylate with a direct photografting of their polymer onto a sensitive gold layer suitable for Surface Plasmon Resonance (SPR) detection [60].

### 2.4. Critical Aspects

Molecular imprinting process presents various advantages, as described above, but some drawbacks must also be considered.

Design of a new MIP system suitable for a specific template molecule often requires a lot of time and work for synthesis, washing and testing. Generally many attempts need to be made, changing various experimental parameters, before finding the optimum conditions. Combinatorial chemistry has been recently adopted in order to accelerate the optimization of MIPs to attain the desired performance [61–63]. Combinatorial approach also allows finding the best MIP composition through the simultaneous synthesis and evaluation of tens or hundreds of imprinted polymers prepared on a small scale (mini-MIPs). Sellergren [63] introduced a high-throughput synthesis and screening (HTS) system in a 96-well plate format that allows rapid optimization and fine tuning of the molecular recognition properties of MIPs library by the use of filter plates for rapid template removal and a multifunctional plate reader for a parallel analysis of the supernatant fractions. In another example, MIP nanoparticles for peptide melittin recognition were obtained using a small combinatorial library of different functional monomers [64]. Only MIPs prepared with proper amounts of *N*-tert-butylacrylamide and acrylic acid showed high affinity for the peptide.

Another important critical point is the design of water-compatible MIPs. For various applications especially in clinical and environmental fields (for instance many biomolecules are insoluble or lose their activities in organic solvents) the imprinting capability of MIPs in water solutions is required. Unfortunately water molecules will compete with the template making weaker or destroying non-covalent interactions (electrostatic, hydrogen and van der Waals bonds) between template and functional monomer. In spite of this, some progress has been made in the development of water-compatible MIPs. Hydrophobic, ionic or metal co-ordination interactions are proving to be very promising to enhance template and functional monomer association in water. Polymerizable-cyclodestrines were used as the hydrophobic part able to control electrostatic interactions between template and monomer from water interferences [65] or mineral oil was added after polymerization to create a hydrophobic shield ables to protect functional groups responsible for molecular recognition [66]. Moreover Piletska *et al*. conducted fundamental research on the physicochemical characterization of a set of imprinted and blank polymers in order to investigate why MIPs, capable of ion pairing, sometimes work in water and sometimes fail. They found that acidic monomers are more effective than basic monomers in creating ionic interactions with the template; secondly, ionic interactions character in MIP can be improved by using strong acidic monomers such as trifluoromethylacrylic acid and, finally, to enhance inclusion of ionized monomers into the polymer, the solvent should have high hydrophobicity [67]. A few studies of imprinting in a mixture of water-containing solvents (methanol/water) such as a porogen have been reported. This choice is done either for solubility reasons, when the template is a polar compound and is not very soluble in solvents, or when the use of a polar solvent favors interactions of the template with the monomers [54,68]. Other papers reported water-compatible MIPs, prepared by introducing hydrophilic properties to the polymer in order to reduce non-specific hydrophobic interactions by using polar porogens [68], hydrophilic comonomers or cross-linkers [69] and/or monomers capable of stoichiometrically interacting with the template functionalities [70].

## 3. MIPs’ Applications

The peculiar properties of MIPs have made them a highly interesting tool for different application areas, including separation sciences and purification, sensors and biosensors, catalysis and drug delivery.

Molecular imprinting chromatography is one of the most extensively studied application areas of MIPs, which are highly suitable for chromatographic separation, allowing the preparation of tailor-made supports with predetermined selectivity. The increasing demand for optically pure compounds makes MIPs for chiral separations another important application especially to obtain the racemic resolution of drugs [22,71]. MIPs have also emerged as new selective sorbents for solid phase extraction (SPE) procedures, allowing not only the preconcentration and cleaning of samples but also the selective extraction of target analytes from complex matrices.

Recently, several studies have demonstrated that MIPs can serve also as artificial binding mimics of natural antibodies and can be used as recognition elements in immunoassay-type analyses. To date MIPs have been successfully used with different types of transducers and several methods have been used to achieve a close integration of the transduction platform with the polymer [24].

Considerable investigations have also been made on the use of MIPs as active materials for catalyzing some reactions. MIPs with catalytic properties can be considered mimics of natural enzymes and applied in enzyme-like catalysis [25,72].

Finally, an area which poses the greatest challenge for MIPs is that of therapeutics and medical therapy. The capability of the MIPs to bind bioactive molecules in specific conditions makes MIT a huge potential for creating suitable dosage forms.

In the next four paragraphs, present and future prospectives of the most important applications will be discussed.

### 3.1. MIPs in Separation Techniques

Molecularly Imprinted Chromatography is one of the most traditional applications of molecularly imprinted polymers [20,33,73] especially for Liquid Chromatography (LC) [74,75] with MIPs usually synthesized by bulk polymerization, ground and sieved mechanically and subsequently packed in a chromatographic column [22]. However, the mechanical processing leads to irregular particles with relatively broad size distribution, resulting in packing of irreproducible quality. For this reason monolithic molecular imprinting columns have been recently prepared directly inside stainless steel columns or capillary columns [76,77]. The monolithic MIPs have fewer nonselective sites than the conventional bulk MIPs particles, even if the polar porogen used for MIPs synthesis can give poorer enantiomeric separation. Many efforts to decrease heterogeneous size distribution have been made also by preparing spherical and monodispersed beads as HPLC stationary phases. Experimental data suggest that not always uniform MIPs particle sizes allow better chromatographic performances. For instance, precipitation polymerization was used to prepare spherical beads but with a total pore volume still lower compared to irregular particles obtained by bulk polymerization and it has been seen that the particles porosity of the beads strongly influences the chromatographic performance of these systems [78]. In fact the calculated pores volume of 0.38 mL/g obtained for beads was lower in comparison to 0.64 mL/g observed for irregular shaped MIPs prepared with similar reagents. LC has been extensively used to evaluate the recognition properties of MIPs since generally it provides a quicker and easier method for analyzing binding polymer performances. Commonly, the retention behavior (k) of the MIP column, is compared with a reference column packed with the Non-Imprinted Polymer (NIP), to evaluate the imprinting effect which is often expressed by the Imprinting Factor (I_f_) calculated as the ratio between the MIP column and the NIP column (k_MIP_/k_NIP_). The MIP sorbents, due to selective interactions exhibited, retain the analyte more strongly than the NIP materials [23].

MIPs were frequently used as Chiral Stationary Phases (MIP-CSPs) in High Performance Liquid Chromatography (HPLC) to obtain enantiomeric resolutions of racemic solutions of different compounds [19], such as amino acid derivatives [74,79–83] and drugs [71,84,85]. The first studies were made by Mosbach group that realized an MIP sorbent used as stationary phase in LC, to separate amino acid derivatives [79]. Recently, Sellergren and co-workers prepared an acrylic polymer by non-covalent imprinting procedure for selective enantioseparation of d or l-Phenylalanine ethyl esters to evaluate the enantio and substrate-selectivity for some amino acid derivates [86]. Yin and co-workers [77] reported studies on the enantioseparation of l-nateglinide by using MIP-CSPs prepared with both bulk and monolithic polymerization process in the same chromatographic conditions. The recognition performances of the monolithic molecular imprinting column showed better performance than the column packed with the polymer obtained by bulk polymerization and this is probably due to the reduction of the number of aspecific binding sites in the monolithic column [87]. However, a limiting factor of monolithic MIP formats is due to the use of porogenic polar solvents required to achieve pore structures of sufficient permeability. The solvent polarity can compete with the template-monomer interactions reducing the affinity of the polymer for the template.

It is worth noting that one of the main drawbacks observed in MIPs for HPLC columns, that limit its commercial application, is the excessive peak broadening and tailing. In fact, they are often found in chromatograms for templates when MIP is used as sorbent and they are attributed to the heterogeneity of the binding sites in the MIPs [33]. More specifically peak broadening and tailing are both thermodynamic and kinetic characteristics. This phenomenon depends on the association constant and sample load, increasing for high values of association constant. Recent efforts to improve chromatographic efficiency have been done and include the replacement of non-covalent imprinting with the covalent one and the use of several strategies for obtaining uniformly sized spherical microspheres.

MIPs have also been used as media for Capillary Electrochromatography (CEC). CEC is a hybrid separation technique that combines the stationary phase of LC with the electrosmotically driven mobile-phase transport of electrophoresis. MIP-based micro-columns for CEC are recently realized for separation of several compounds [73,88,89].

Solid Phase Extraction (SPE) is another important area of application of MIPs in analytical chemistry [21,23,90–93]. MIP for Solid-phase extraction (MISPE) has been applied both in on-line and off-line procedures. MIPs particles, used as selective sorbent materials, can be packed in an HPLC precolumn for the on-line mode and in a cartridge between two frits for the off-line mode [14]. The on-line MISPE procedure, is directly coupled with specific analytical systems such as HPLC, minimizing samples manipulation and reducing the loss of analytes and the risk of contamination [92,94]. Furthermore, this method considerably reduces the time for pretreatment of the samples. Despite these advantages, the off-line method is the most used for its simplicity. Moreover, more solvents can be handled without regard to their influence on the successive separation methods.

**Figure 6:**
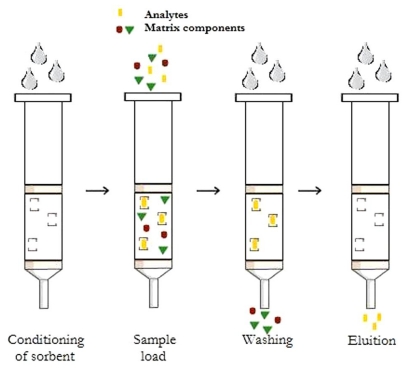
The four steps of the MISPE process.

The principle of MISPE is based on the same main four steps as classical SPE: conditioning of the sorbent, loading of the sample, washing away interferences and elution of the target analytes (Figure 6). In the loading step, the sample is percolated through the MIP sorbents. Generally, this solvent must have a polarity similar to that used in the polymerization process, since it increases the number of interactions between analyte and specific binding sites in the MIP sorbents [46].

MISPE was applied for extraction of several compounds in different sample matrices such as biological [54,95–104], environmental samples [15,105–111] and also in food analysis [16,112–117].

The first application of MISPE was made by Sellergren in 1994 which prepared a selective extraction of pentamidine, a drug used for the treatment of AIDS-related disorders, in urine samples [95]. The pentamidine-imprinted materials were prepared by *in situ* polymerization in a chromatographic column coupled with a simple HPLC system.

**Figure 7:**
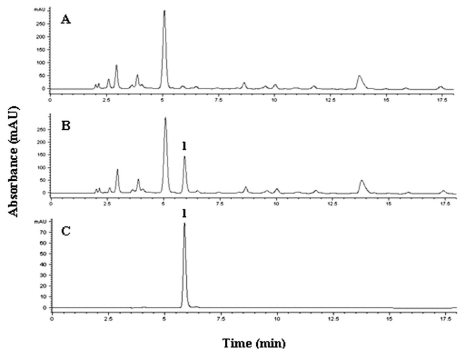
Chromatograms corresponding to (**a**) human urine; (**b**) human urine spiked with 1-MA; and (**c**) elution solution after MISPE of spiked urine. Peak identification:1, 1-MA (Adapted from [54]).

Recently, some MISPE for vitamins and nucleoside, derivatives from biological matrices, were prepared. A highly selective MIP for 1-methyladenosine (1-MA), an urinary modified nucleoside which is used as cancer marker, has been prepared and used as sorbent material in an off-line SPE mode [54]. The polymer has been obtained by bulk polymerization using MAA as functional monomer and a mixture of acetonitrile/water (4:1, v/v) as porogen, to overcome the problems related to the imprinting of biological compounds. As can be seen from Figure 7, 1-MA has been selectively extracted by the MIP polymer after removing the interfering compounds present in the urine.

In another study [96] a selective molecularly imprinted polymer, obtained by bulk polymerization technique, was prepared as sorbent for solid phase extraction of nicotinamide. Moreover, the performances of the imprinted polymer have been compared to that of non-imprinted polymer and with conventional reversed-phase C18 performances and have shown better results (Table 1).

Javanbakht and co-workers have recently studied the extraction of important drugs from biological samples, such as plasma and urine. They followed a new water-compatible methodology for determination of the analgesic Tramadol from biological fluids, using MISPE as the sample clean-up technique combined with HPLC [97]. The water-compatible imprinted polymer was prepared using MAA as monomers and chloroform as porogen; loading the sample with water at pH 8 and using methanol/acetic acid (10:1, v/v) as elution solvents. The results showed that the recoveries of Tramadol for the spiked human plasma and urine samples were higher than 91% in the range of 10–50 μL^−1^. The same methodology was used to extract other drugs from plasma and urine, such as Verapamil, an L-type calcium channel blocker [98] and Bromhexine, a mucolytic agent [99].

MISPE procedure combined with HPLC was also applied to extract Testosterone and Progesterone and other important drugs from urine [100,101,104].

In recent years, MISPE was extensively applied also for the determination of analytes in environmental samples. In particular, phenolic compounds were widely studied for their pronounced toxicity and their wide distribution in several environmental samples. In the last few years, a new class-selective sorbent for SPE to pre-concentrate the environmental pollutants, phenolic compounds, from spiked water samples was developed by Peipei *et al.* [105]. A MIP was prepared by bulk method using 2,4-dimethylphenol, that has been chosen, because of its low toxicity, as template. Synthesis optimization was done by using three different porogens, chloroform, acetonitrile and toluene and the difference in recognition selectivity of the polymer columns was observed in HPLC system. The SPE methodology was then developed and used to preconcentrate phenolic compounds in the environmental water. The resulting MIP showed good selectivity for 2,4-dimethylphenol, 2,4-dichlorophenol, 4-chlorophenol, 4-methylphenol and phenol. The application of MIP-SPE to environmental water samples confirms the ability of the MIP sorbent to class-selectively isolate phenolic compounds from the complex matrix.

On-line MISPE coupled to reversed-phase HPLC was also applied to selective extraction of 4-nitrophenol in real water samples [106]. MISPE was also applied for the extraction of herbicides, so, several MISPEs for Triazine-based herbicides have been reported for different food matrix such as vegetables and fruits, involving extraction of different analytes such as simazine [112], propazine [113], propazine methacrylate [114], *etc*. From the examples reported above we can say that MISPE is a competitive analysis procedure compared with traditional solid-phase extraction for the stability and low cost of preparation of the imprinted materials. Today there are already MISPE cartridges commercially available for selective extraction of several molecules [112,118] which will increase MISPE applications in analytical chemistry.

It is well known that template residues are present in the polymer matrix even after exhaustive washing steps and leakage of the template can affect the accuracy of detection in trace analysis. To overcome this bleeding problem the MIP can be synthesized by using a close structural analogue of the analyte as template (also called dummy template) that can capture also the target analyte since very similar cavities are generated [119,120]. According to a recent work [120], a MIP for 5-nitroimidazole recognition was prepared using a template analogue which was different from the analytes studied, methacrylic acid as monomer and divinylbenzene as cross-linker in chloroform. For the first time a MISPE protocol was developed for the selective extraction of four different 5-nitroimidazoles and three of their metabolites in egg powder. The 5-nitroimidazoles and their deuterated analogues showed similar and good behavior on the MISPE cartridges with satisfactory linearity at the concentration studied. The validated method provided enough accuracy, reproducibility and sensitivity. In the real matrice, the lack of real internal standard was observed for two analytes while the others gave recovery higher than 91%.

Several studies applying imprinting technology to different selective extraction techniques have appeared over the last few years such as Solid-Phase Microextraction (SPME), Stir-Bar Sorptive Extraction (SBSE) and Matrix Solid Phase Dispersion (MSPD) techniques.

The first work on SPME appeared in 1990 [121] and, since then, many different studies have looked at using this simple method to extract different analytes from many different matrices. Firstly the fibers were synthesized and then MIPs were used as sorbents on those fibers by using a bulk polymerization method. In this case, the monolith obtained from traditional polymerization is attached to a solid support. A suitable silylation of the support is important for this technique, but there is no standardization so far on the fibers preparation step and there are several different supports described in literature to attach the fibers. The most widely-used support has been a commercial fiber in which the coating surface has been etched away [122,123]. For instance, Prasad *et al*. extracted ascorbic acid from human serum with recoveries of 100% without any sample pretreatment. Another way to obtain SPME fibers is, by using a glass capillary, filling it with the polymerization mixture and inducing the polymerization process inside [124,125]. Another support that can be used is a hollow tube, such as an optic fiber, to which the MIP is attached [126]. A common limit for most of these fibers is their fragility since they are formed by highly cross-linked monolithic polymer.

MIPs have been used as coating agents also for Stir-Bar Sorptive Extraction (SBSE) technique [127–129] first introduced by Baltussen *et al.* in 1999 [130] with the main purpose of increasing the mass of sorbent used in SPME fibers. This technique is based on the use of a magnet covered of polydimethylsiloxane which is able to retain some compounds present in solution. Desorption of the extracted compounds is performed using very low volumes of organic solvent or thermally in gas chromatography equipment. For instance, Hu *et al.* [129] selectively extracted four extrogens from water samples. They used an iron bar held inside a glass tube and sililated the outer surface of the glass. Then the glass was put inside a mixture of polydimethylsiloxane, β-cyclodextrin as the template molecule and all the typical compounds involved in the synthesis of a normal stir-bar and a molecularly imprinted polymerization. This step was repeated several times until the desired thickness of the stir-bar was achieved.

Matrix Solid-Phase Dispersion (MSPD), commonly used for solid samples, is another extraction technique which differs from the previous ones mainly since the extraction can also be performed directly on solid, semisolid and viscous samples. In this technique the sample of interest and the sorbent are blended together; then, the obtained mixture is packed into an empty column or a SPE cartridge or a syringe. Finally, the analytes can be extracted through two different ways depending on their affinity towards the sorbent used. When there is a high retention of the analyte of interest on the sorbent, a solvent can be added into the cartridge to remove the interfering compounds; while if the analyte of interest is not strongly retained on the sorbent, a solvent able to disrupt only the weaker interactions can be added to directly remove the analyte. In MSPD extraction method, any commercially available sorbent can be used. Some authors have also tried to combine the advantages that MIPs offer for selective extraction with the simplicity of the present technique [131,132]. Guo *et al*. [131] determined a chloramphenicol amount in fish tissue samples with higher recoveries than any other commercially available sorbent.

### 3.2. MIPs as Chemical Sensors and Biosensors

In the last years, chemical sensors and biosensors are of increasing interest in the field of modern analytical chemistry, as can be seen from the growing number of published papers. This is due to new demands that are appearing particularly in clinical diagnostics, environmental analysis and also in food analysis. Recently, a big effort to synthesize artificial receptors capable of binding a target analyte with comparable affinity and selectivity to natural antibodies or enzymes has been done.

MIP technology can also be used as antibody-like materials with high selectivity and sensitivity, owing to their long-therm stability, chemical inertness and insolubility in water and most organic solvents [133]. To date, MIPs have been successfully used with different types of transducers and several methods have been used to achieve a close integration of the transduction platform with the polymer [24]. In particular, the integration of MIPs with sensors can be realized by *in situ* polymerization, using a photochemical or thermal initiator [134], or by surface grafting with chemical or UV initiation [135,136]. The advantage of this latter approach lies in the possibility of controlled modification of inert electrode surfaces with thin films of specific polymers.

Moreover, polymers can also be electropolymerized on the surface of transduction platform. In this case, there are some adhesion problems, especially during the washing process of the polymerized MIP-coated QCM (Quartz Cristal Microbalance) with organic solvents that, sometimes lead to a partly peeling off of the MIP layers produced. For this reason, specific pretreatment to enhance the adhesion of MIPs on transducer platforms, must be done.

The first generation of MIP sensors was prepared using imprinted polymers synthesized in the form of monoliths. The obtained MIP particles were deposited in close proximity to the electrode by incorporation of the particles into the carbon paste of screen-printed electrode [137] or into a supporting agarose gel [138]. It was observed that the response of the sensor was strongly dependent on how intimately the MIP and transduction element were integrated and how efficient the electrical communication was between them.

Competitive amperometric morphine sensor using MIP particulates immobilised with agar gel on the electrode surface, has been reported by Mosbach’s group [139]. The device employed a competitive two-stage displacement strategy in which initially the electro-active template bound to a morphine imprinted polymer which had been immobilized in a thin layer of agarose. Subsequently an electro-inactive competitor, codeine, was added which resulted in the displacement of the morphine from the MIP. The displaced template was then determined amperometrically. The result obtained in this study reported a working concentration range of 0.1–1.0 μg/mL and also a stability of the sensor in a range of different conditions. In particular these results suggest a high degree of ruggedness.

MIP-based recognition elements were also prepared as layers or thin films by deposition or grafting onto the transducer platform [140,141]. The thickness of the film deposited on the transducer is important to obtain a good time response of the sensor. This approach was firstly used with acoustic [142] and optical [143] transducers and then with electrochemical sensors [144].

Today, a simple approach to obtain sensor device, is the combination of MIPs with a piezoelectric transducer (e.g., quartz) to create an acoustic sensor called QCM-MIP sensor. The coating of the crystal with the MIP can be obtained by *in situ* polymerization directly onto the surface of the device [145,146] or via pre-prepared MIP particles that are immobilized on the sensor surface using a PVC matrix [147].

A QCM-MIP sensor has been employed by Peng and co-workers to obtain a sensor device for atropine detection. In this case, the electropolymerization approach was used to prepare an aniline-co-*O*-phenylenediamine atropine MIP layer onto a piezoelectric quartz crystal to realize a bulk acoustic-wave sensor. The MIP sensor obtained was successfully employed to determine atropine concentrations in human serum and urine [148].

Moreover, a sorbitol-based MIP was also prepared by electropolymerization of o-phenylenediamine on a QCM surface [149]. Over a concentration range of 0–16 mM the resulting sensor showed high selectivity for sorbitol, compared with glucose, glycerol, mannitol and fructose.

In another study, an imprinted polymer-coated sensor was created by Tan *et al.* to determine the amount of paracetamol and nicotine in biological fluids [150,151]. In this case, piezoelectric quartz crystal surface was modified using a paracetamol imprinted polymer as sensing materials to obtain selectivity for a Bulk Acoustic Wave (BAW) sensor. The kinetic impedance analysis showed no change in the viscoelasticity of the polymer coating during the detection. Satisfactory results have been obtained in the recognition of paracetamol in human serum and urine.

Recently, Ersőz and co-workers have developed a new QCM sensor for specific determination of 8-hydroxy-2′-deoxyguanosine (8-OHdG), an oxidative stress marker [152]. The MIPs was prepared by *in situ* polymerization of methacryloylamidohistidine-platinum(II) and *N*-*N*′-methylenebisacrylamide on QCM surface and used to determine the amount of (8-OHdG) in the blood of cancer patients. QCM-MIP sensor was also realized for detection of daminozide, a potential carcinogenic chemical important in food safety [5] but also for detection of environmental pollutants such as bisphenol A [153], acetaldehyde [153] and monoterpenes [154].

MIPs were also widely employed as sensors for enantiomeric separation of different compounds such as R and S-propranolol, d and l-tryptophan, and d and l-serine enantiomers [147,155,156]. Several efforts to adapt MIP-based QCM sensing technology to chiral recognition of (*S*)-propranolol have been reported by Haupt and co-workers that created an enantioselective chiral recognition layers on the gold-coated surfaces of 5 MHz quartz crystals employing a poly(TRIM-*co*-MAA) MIP formulation [147]. To ensure the formation of homogeneous MIP coatings and to minimize inhibition of polymerization by oxygen, the imprinting mixture was sandwiched between the QCM and a quartz cover plate before polymerization. This sandwich exposed to UV irradiation produced MIP films 2 μm thick with good adherence to the supporting resonator surface. The results obtained showed a pronounced decrease in the resonator frequency for (*S*)-propranolol over the analyte concentration range investigated (0.05 to 1.5 mmol L^−1^) whereas (*R*)-propanolol induced only minor frequency changes.

However, in terms of sensibility, MIP-based biomimetic sensors are still rather inferior to biosensors because they require further optimization of the MIPs and the transducers. An interesting area of research was the preparation of mass sensitive MIP sensors for cells and viruses. In particular, Dickert and co-workers realized a QCM-MIP sensor able to bind with excellent specificity the yeast *Saccaromyces cerevisiae* used as template in the imprinting process [157]. The same authors used a similar approach to obtain a molecularly imprinted polymer sensor for tobacco mosaic virus and red blood cells [158,159].

During the last decade, the MIP technology has been extensively studied for its potential applications in pseudo-immunoassay [28]. In particular, the first Molecularly Imprinted Sorbent Assay (MIA) was reported by Mosbach for detection of theophylline in serum in comparison with the commercial Enzyme-Multiplied Immunoassay Technique (EMIT) [160]. This first MIA showed an excellent correlation (correlation coefficient = 0.98) with EMIT, and also produced a strikingly similar cross-reactivity profile to that of natural antibodies. Although this MIA method was found to be more cumbersome, it was cheaper and faster than that of natural antibodies. Moreover, MIPs have demonstrated remarkable stability under storage in dry state at ambient temperatures, surviving for several years without loss of recognition capability.

The competitive formats of MIAs can be divided into two categories: homologous and non-homologous assays, in which analyte and probe are chemically identical or chemically different respectively. Radio-labeled MIAs were the first examples of homologous assays where the probe is just the radio-labeled form of the analyte used as template in the imprinting process. In particular, novel imprinting processes to create spherical, molecular imprinted beads were employed in radio-labeled MIAs as alternatives to conventional MIP particles preparation with several advantages such as enhanced selectivity, low cross-reactivity and a simple bead preparation.

Recently, Ye *et al.* synthesized (*R*,*S*)-propranolol imprinted microspherical beads by a modified precipitation polymerization and they were used in competitive radio-labeled MIAs for chiral analysis of propranolol, using (*S*)-[4-^3^H]Propranolol as radioactive probe. When the MIP was used in scintillation proximity assays, the binding of the radio-labeled template determined a transfer of energy from template to scintillant resulting in the generation of a fluorescence signal [161]. In another study, 17β-estradiol imprinted beads prepared by precipitation polymerization were employed in a competitive radio-labeled MIAs to obtain a stereoselectivity for 17β-estradiol over its diasteroisomer 17α-estradiol [78].

Nevertheless, the practical applications of this technology are heavily restrained due to the inherent complications of handling and disposing of radioactive materials. For this reason, fluorescent analogues of analytes were employed as alternative probes in MIAs. Conventional fluorescent-labeled MIAs is an example of non-homologous assay where the MIP is prepared using the analyte itself as template in the imprinting process and a fluorescent probe in place of the radioactive probe. However, a problem that hinders the development of this technique is due to different structures of the fluorescent probe, which has additional fluorescent groups to that of the analyte, inducing a decrease of sensitivity and selectivity. Nevertheless, in the last few years, many researchers have focused their interest on fluorescence-labeled MIAs [162–165] primarily for their attractive practical features in future applications.

### 3.3. MIPs in Catalysis

Considerable efforts have been made to investigate the possible use of MIP for catalytic applications [25–72]. The high selectivity and strength of these polymers make them suitable to be used at elevated temperatures and pressures, in the presence of several organic solvents, and also under acidic and basic reaction conditions. For these reasons, MIPs can be employed instead of biomolecules, such as enzymes and natural catalytic antibodies [166] which are highly vulnerable to certain conditions.

The use of MIP for catalytic application is very important because MIP catalysts are able to mimic the selectivity and sterospecificity of the binding domains of antibodies and enzymes which are generally utilized as catalysts in several reactions. Catalytically active imprinted materials can be obtained using analogues of substrates, transition states or products as templates in the imprinting protocol [29]. The polymer matrix obtained has cavities with a shape similar to the shape of the template used [72,167]; but the imprinting technology must also ensure the right placement of the functional groups in the binding sites in consideration of the type of catalytic process that they will assist [168].

**Figure 8:**
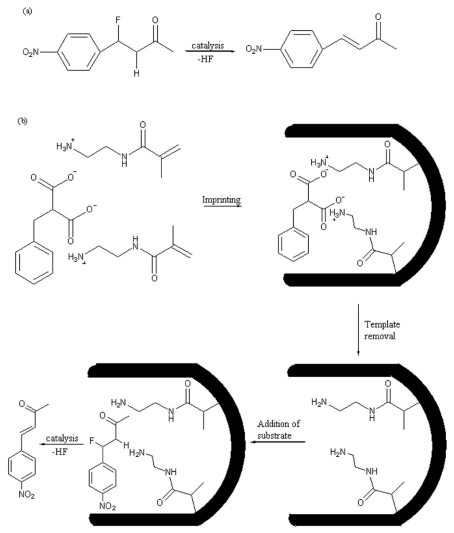
MIP catalytic systems developed by Beach and Shea (Adapted from [170]).

The strategy using substrate analogues as template, involves the use of compounds that mimic the reaction complex between the substrate and the matrix. The catalytic groups will be introduced in the right positions into the cavities of the polymer and subsequently they will act catalytically in presence of the true substrate. Leonhardt and Mosbach applied this strategy to obtain an imprinted matrix with esterolytic activity using Cobalt (II) ions to coordinate vinylimidazole groups and template during polymerization. Subsequent introduction of the substrates, such as p-nitrophenyl esters of methionine or leucine, into the cavities of polymer, leads to an acceleration and substrate-specific hydrolysis of amino acid analogues [169]. The same strategy was applied by Shea and Beach to obtain a dehydrofluorination of β-fluoroketones using *N*-(2-aminoethyl)mathacrylamide as basic functional monomer with catalytic groups (Figure 8). Several dicarboxylic acids were employed as template analogues to obtain the correct positioning of the amino groups in the cavities of the polymer. It has been seen that the catalytic activity of the polymer mainly depends on the position of carboxylic groups of the template. Benzylmalonic acid has proven to be the best template to obtain the most active imprinted polymer [170].

An alternative approach to obtain the same results was the use, in the polymerization process, of a basic Transitional State Analogue (TSA) and MAA respectively as basic template and acidic functional monomers. The use of the MIP catalyst in the reaction process led to an increase in dehydrofluorination rate compared with the uncatalyzed reaction. The TSA approach was followed by Wulff and co-worker to obtain a phosphonate ester imprinted polymer using amidines as monomers. The amidines have the ability to bind carboxylic acids and phosphonate esters with very high affinity, such that the complexes are almost completely in the associated form, even in water. The catalyst was tested on the hydrolysis reaction of analogous carboxylate esters. High catalytic activity was seen, despite the nonactivated nature of the esters. The reaction rate was 100 times higher for the imprinted matrices compared with the uncatalysed solution reaction [171].

Very recently, Li *et al.* [172,173] prepared smart imprinting systems with catalytic activity. A catalytic and positively thermoresponsive MIP was prepared for the first time and used for catalytic hydrolysis of 4-nitrophenyl acetate, used as model reaction. At a relatively low temperature, such as 20 °C, the polymer did not show significant catalysis due to the interpolymer complexation that causes a shrinking of the system and blocks the access to the active sites. On the contrary, at higher temperature, such as 40 °C, the MIP gave significant catalytic activity [172]. In another work, Li *et al.* reported a smart like-enzyme imprinted polymer composed of poly(*N*-isopropylacrylamide)-containing p-nitrophenyl phosphate-imprinted networks. In this case, at a relatively low temperature, such as 20 °C, the polymer showed vigorous catalysis due to its hydrophilic network that enables the access to the active sites while at higher temperatures, a significant decrease of MIP hydrophilicity causes shrinking of the imprinted polymer which inhibited its catalytic activity [173].

Finally, thermodynamic and kinetic studies were also carried out on MIPs catalytic systems with the aim of understanding molecular imprinting and its specificity [174]. The p-nitrophenyl phosphate-imprinted polymer was prepared, characterized and used as catalyst for a model hydrolysis reaction. Kinetic studies indicate that the specific mechanism from imprinted polymer catalysts can be internally due to the larger speedup for the imprint species. The reaction rate of the imprint species can be much higher than that of its analogue. Thermodynamic study also implies that the induction generated from the specific imprint to the imprint species is stronger than to its analogue. Thus, the increasing rate and the larger induction, in logic, may be responsible for the specific recognition and catalysis.

### 3.4. MIPs in Drug Delivery

The ability of MIPs to bind strongly and selectively bioactive molecules makes these materials suitable for their potential application in biological field. The high loading capacity and the prolonged release time of the analytes, such as drugs, amounts to MIPs having a huge potential for creating suitable dosage forms [26].

In the last years, an area of great challenge in MIP technology is that of therapeutic agents, various MIPs have been used as unusual synthetic polymeric carriers to prepare drug delivery systems [175,176].

An efficient drug delivery system should ensure that the drug is released at the right site, in the right dose and for the right period of time [177].

MIPs for drug delivery applications should have specific characteristics: the imprinted cavities should be stable to maintain the conformation in the absence of the template, but also flexible to facilitate the realization of a fast equilibrium between the release and re-uptake of the template in the cavity. To this end, the non-covalent imprinting provides faster equilibrium kinetics than the covalent imprinting approach [178,179]. Furthermore, MIPs should be stable to resist enzymatic and chemical attacks and mechanical stress that can be found in biological fluids.

Imprinted-drug delivery systems have not reached clinical applications yet because the adaptability of molecular imprinting technology for drug delivery requires important remarks on safety and toxicological concerns since the polymer devices will go into contact with biological tissues without causing toxic effects [180]. The MIPs are normally synthesized in organic solvents to improve hydrogen bonding and electrostatic interactions [178]. Nevertheless, the presence of organic solvents, commonly used in MIPs synthesis, may cause cellular damages. For this reason, in drug delivery processes, it is usually advantageous to prepare hydrophilic polymers which are compatible with biological systems. Alternatively precipitation polymerization method can be used since the polymer is usually completely immiscible with the solvent used for MIP preparation allowing easy polymer separation from the solvent [176]. Molecular imprinting water compatible is still under development and there are many problems due to the considerable weakness of hydrogen bonding and electrostatic interactions in water that decrease the selectivity of the MIP for the target molecule. However, metal coordination and hydrophobic interactions can be exploited to enhance template and functional monomer interactions [65,181].

Recently, molecularly imprinted hydrogels for the recognition of cholesterol have been prepared by molecular design of methacrylate-based structures containing poly(ethylene glycol) in moderately and highly cross-linked networks. Swelling studies have been done to analyze the cross-linked structures of the prepared hydrogels and recognitive studies have been carried out to evaluate the influence of different reaction conditions on the ability of the systems to absorb cholesterol. The imprinted hydrogels have shown an excellent ability to recognize and release the analyte of interest [182].

In recent years, many researchers have published about the use of MIPs in drug delivery applications [179,183] given their physicochemical properties to protect the drug from degradation by enzymes during systemic trafficking in the body. Several drugs have been used as templates to achieve polymeric devices, capable of prolonging the release profile of specific therapeutic agents with better performance as compared to more traditional drug delivery system [175].

The first report of imprinted polymers used as sustained release devices has been presented by Norell and co-workers [184]. Theophylline-imprinted polymer has been prepared by a non-covalent approach for its possible application as a controlled release. The polymer obtained was able to sustain a slow drug release in pH 7.0 phosphate buffer for several hours. Allender *et al.* prepared MIPs for Propranolol (a β-adrenergic antagonist) using methacrylic acid (MAA) as a functional monomer to create a transdermal controlled release device and they demonstrated that the permeation of Propanolol was slower for the MIP than from the non-imprinted polymer. Thus, these Propranolol-imprinted systems can be used to prolong the delivery profile [185]. Similar results were obtained with other drugs, such as tetracycline [186] and sulfasalazine [187]. Cai and Gupta [186] using tetracycline as template synthesized MIPs with higher binding ability compared with corresponding non-imprinted polymers. The obtained results indicated that the high MIP affinity can be utilized in control release applications, in fact the tetracycline release from MIP was slower than non-imprinted polymer. In another study [188] a molecularly imprinted polymer enables to rebind nicotinamide selectively and release it in a sustained way was prepared. MIPs microspheres have shown also a higher binding properties and sustained release compared to the non-imprinted one. This polymer could represent a promising system in the preparation of nicotinamide controlled formulations for oral administrations. In order to get controlled release of nicotinamide, various MIPs have been synthesized with methacrylic acid and ethylene glycol dimethacrylate by precipitation polymerization. It has been found that the use of different stoichiometric ratio between template, monomer, and cross-linking agent influences binding and release properties of the polymer.

Intelligent drug release, refers to the release of a therapeutic agent, may occur as a result of a specific stimuli such as the presence of another molecule. A system with similar characteristics, able to release testosterone at a rate depending on the concentration of hydrocortisone, was described by Sreenivasan *et al.* In this study, hydrocortisone as template was used for the preparation of MIP. After the removal of target analyte, the MIP was loaded with testosterone, which is a structurally similar molecule. It was seen that the rate of testosterone release increased when hydrocortisone was present in the solution as a result of template responsive release [189].

Molecularly imprinted polymers can be used also to bind several substances in the gastrointestinal tract, blocking their absorption in the body, as a complement of the pharmacological therapy. In this contest, MIPs as chemical traps to remove undesirable substances from the body, such as glucose, cholesterol [182,190,191], bile acid [192] and melittin [193] were developed. Hoshino and coworkers prepared imprinted polymers nanoparticles able to efficiently capture the cytotoxic peptide melittin in the bloodstream, synthesizing protein-size polymer particles with a binding affinity and selectively comparable to those of natural antibodies, by combining MIP nanoparticle synthesis with a functional monomer optimization strategy [193].

## 4. Conclusions

This review has attempted to outline various aspects of molecular imprinting technologies. All considered features present strengths and weaknesses and for any specific application some advantages or problems need to be evaluated.

Important progress in the synthesis and application of MIPs has been reported consistently in the literature within recent years. Low cost, excellent stability and continuously advancing performance of MIPs make these polymers the most promising synthetic materials for molecular recognition in different scientific fields.

New procedures of alternative syntheses for bulk polymerization that can improve the properties of MIPs particles, should promote future development of MIPs in different application fields.

MIPs have been widely studied as HPLC stationary phases, especially for chiral separation. However, in this field one aspect that would enhance performance would be to overcome the broadening and tailing of the template peak that is often observed.

More recently, an increasing number of papers on MISPE have been produced. MISPE is a competitive analysis procedure for the stability and low cost preparation of the imprinted materials, compared with traditional solid-phase extraction. Today there are already MISPE cartridges commercially available for selective extraction of several molecules [112,118].

In recent years, particular attention has been directed to the development of MIPs as selective material for sensors and biosensors. However, in terms of sensibility, MIP-based biomimetic sensors are still rather inferior to biosensors because they require further optimization of the MIPs and the transducers. Thus, although the stimulating ongoing work in this field, the commercial development of molecular imprinting sensors is still in its infancy.

Catalysis and drug delivery applications have not yet been thoroughly investigated. Applications in these areas are only just beginning to emerge and there are several potential aspects to be explored that could produce progress in the next few years.

## Figures and Tables

**Table 1:** Recoveries of nicotinamide in spiked pork liver samples using different SPE sorbents (*n* = 3, spiked level, 49 μg mL^−1^) (Adapted from [96]).

SPE sorbent	Recovery (%)	RSD (%)
N-MIP	87	8
NIP	12	5
RP C18	14	6
